# The Effect of Polyethylene Glycol on the Non-Isothermal Crystallization of Poly(L-lactide) and Poly(D-lactide) Blends

**DOI:** 10.3390/polym16152129

**Published:** 2024-07-26

**Authors:** Panthima Phuangthong, Wenwei Li, Jun Shen, Mohammadreza Nofar, Patnarin Worajittiphon, Yottha Srithep

**Affiliations:** 1 Manufacturing and Materials Research Unit, Department of Manufacturing Engineering, Faculty of Engineering, Mahasarakham University, Mahasarakham 44150, Thailand; 65010351503@msu.ac.th (P.P.); liwenwei918@gmail.com (W.L.); shenjun880212@gmail.com (J.S.); 2 College of Mechanical Engineering, Hunan Mechanical & Electrical Polytechnic, Changsha 410151, China; 3 Sustainable & Green Plastics Laboratory, Metallurgical & Materials Engineering Department, Faculty of Chemical and Metallurgical Engineering, Istanbul Technical University, Istanbul 34469, Turkey; nofar@itu.edu.tr; 4 Department of Chemistry, Faculty of Science, Chiang Mai University, Chiang Mai 50200, Thailand; patnarin.w@cmu.ac.th; 5 Center of Excellence for Innovation in Chemistry (PERCH-CIC), Faculty of Science, Chiang Mai University, Chiang Mai 50200, Thailand; 6 Center of Excellence in Materials Science and Technology, Chiang Mai University, Chiang Mai 50200, Thailand

**Keywords:** poly(L-lactide), poly(D-lactide), stereocomplex, polyethylene glycol, crystallization kinetics, non-isothermal crystallization

## Abstract

The formation of polylactide stereocomplex (sc-PLA), involving the blending of poly(L-lactide) (PLLA) and poly(D-lactide) (PDLA), enhances PLA materials by making them stronger and more heat-resistant. This study investigated the competitive crystallization behavior of homocrystals (HCs) and stereocomplex crystals (SCs) in a 50/50 PLLA/PDLA blend with added polyethylene glycol (PEG). PEG, with molecular weights of 400 g/mol and 35,000 g/mol, was incorporated at concentrations ranging from 5% to 20% by weight. Differential scanning calorimetry (DSC) analysis revealed that PEG increased the crystallization temperature, promoted SC formation, and inhibited HC formation. PEG also acted as a plasticizer, lowering both melting and crystallization temperatures. The second heating DSC curve showed that the pure PLLA/PDLA blend had a 57.1% fraction of SC while adding 5% PEG with a molecular weight of 400 g/mol resulted in complete SC formation. In contrast, PEG with a molecular weight of 35,000 g/mol was less effective, allowing some HC formation. Additionally, PEG consistently promoted SC formation across various cooling rates (2, 5, 10, or 20 °C/min), demonstrating a robust influence under different conditions.

## 1. Introduction

The depletion of fossil fuels and the waste generated by nonbiodegradable plastics are two sustainability and environmental issues that can be mitigated by the use of bio-based polymers [1,2]. Polylactide (PLA) is a promising eco-friendly polyester material that offers numerous advantages over conventional petrochemical polymers [3]. Derived from natural resources such as corn, wheat, and sorghum, PLA serves as a sustainable alternative to petroleum-based plastics [4]. PLA is both biocompatible, meaning it is safe for use in contact with biological systems, and biodegradable, breaking down into harmless byproducts under appropriate conditions [5]. Furthermore, high-molecular-weight PLA exhibited excellent mechanical properties and thermoplasticity comparable to traditional plastics. Despite its many benefits, PLA faces challenges related to its low crystallinity and slow crystallization rate, which can limit its suitability for certain applications requiring high mechanical strength or rapid processing [6,7,8]. Blending PLA with other materials is an effective approach to enhance and tailor its mechanical and thermal properties [9,10,11].

**Figure 1:**
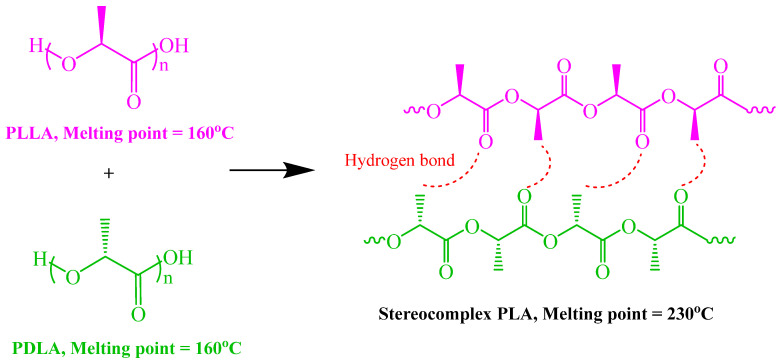
Enantiomeric PLA homopolymers blend to form polylactide stereocomplex (sc-PLA).

PLA has two enantiomeric forms: poly(L-lactide) (PLLA) and poly(D-lactide) (PDLA), which are mirror images of each other. When blended, PLLA and PDLA can form a unique structure known as a stereocomplex crystal (SC) [12,13,14]. Rathi et al. [15] and Tsuji et al. [16] found that the polylactide stereocomplex (sc-PLA) typically exhibits enhanced mechanical properties, such as higher stiffness and heat resistance, compared to the individual PLLA and PDLA polymers. This makes sc-PLA a desirable material for various applications, including biodegradable plastics, biomedical devices, and packaging materials. The melting temperature (T_m_) of the sc-PLA is approximately 230 °C, significantly higher than that of individual PLLA or PDLA, indicating enhanced thermal stability [17]. However, achieving a high concentration of stereocomplexes in PLA is challenging. This is because the HC and SC of each enantiomer (PLLA and PDLA) occur simultaneously and compete with one another [18,19]. Figure 1 illustrates the stereochemical structures of PLLA and PDLA as they combine to form a stereocomplex. This formation occurs through hydrogen bonding between the methyl hydrogen of one PLA compound and the carbonyl oxygen of the corresponding enantiomeric PLA compound [20].

Poly(ethylene glycol) (PEG) serves as a commonly used plasticizer for polylactide (PLA) polymers. Researchers have explored various characteristics of PEG, such as the molecular weight (M_w_), polarity, and end groups, in order to enhance its plasticization efficiency and customize its impact on PLA properties [21,22,23,24]. Kodal et al. [25] investigated biodegradable polymer blends comprising poly(L-lactide) (PLLA) and PEG, specifically focusing on PEG molecular weights between 400 and 35,000 g/mol. In a related study, Takhulee et al. [26] suggested that PLA and PEG might demonstrate miscibility at low PEG concentrations (10–30 wt%). Moreover, based on the information from Bao et al. [27], incorporating PEG with a molecular weight ranging from 1000 to 6000 g/mol into a concentration of 2 to 10 wt% enhanced the crystallinity of PLLA/PDLA blends during melt solidification. Both the concentration and molecular weight of PEG influenced the crystal structure of the blend. Specifically, SC crystallized more rapidly with lower-molecular-weight PEG or higher concentrations of PEG. However, further research specifically focusing on the non-isothermal crystallization of PLLA/PDLA blends with varying PEG content and molecular weight is needed. This area remains understudied despite the observed effects on crystallinity in melt-solidified conditions.

In isothermal crystallization, the material is held at a constant temperature to allow crystallization to occur, providing insight into the crystallization kinetics under controlled thermal conditions. In contrast, non-isothermal crystallization involves cooling the material at a constant rate, which more closely simulates industrial processing conditions and provides information on how the material crystallizes under dynamic thermal conditions. Common processing techniques such as extrusion, injection molding, and film casting entail cooling the molten PLA to trigger crystallization and solidification. Through investigations into the non-isothermal crystallization kinetics of PLA-based systems, researchers can fine-tune the processing conditions to attain the desired properties in the end product [28]. Considerable research has delved into the non-isothermal crystallization kinetics of PLA-based systems, encompassing PLA with various chemical structures and compositions [29,30]. Moreover, studies have shown that shear flow plays a crucial role in polymer crystallization, affecting nucleation and crystallinity in materials like PLA. For instance, Gao et al. [31] investigated shear-induced crystallization in injection-molded poly(L-lactic acid) (PLA) using vibration-assisted molding techniques. Their findings highlighted that intense flow fields could promote the formation of oriented crystalline structures, significantly enhancing both the crystallinity and mechanical properties of PLA.

The novelty of our manuscript lies in quantifying the effect of PEG on sc-PLA crystallization of 50/50 PLLA/PDLA blends and demonstrating this through non-isothermal crystallization kinetics. In this study, PEG with molecular weights (M_w_) of 400 g/mol and 35,000 g/mol at concentrations ranging from 5% to 20% by weight were used as plasticizers for 50/50 PLLA/PDLA blends. We quantitatively investigated the effect of different molecular weights of PEG on the crystallization of sc-PLA in PLLA/PDLA blends at various concentrations. The kinetics of non-isothermal crystallization of sc-PLA was studied at different cooling rates to elucidate how the presence of PEG plasticizer affects the rate and extent of crystallization. This comprehensive analysis provides a deeper understanding of PEG’s role in the crystallization process, highlighting its impact on both crystallization temperature and the formation of SCs, and offers insights into optimizing the material properties of PLLA/PDLA blends.

## 2. Experimental

### 2.1. Materials

Poly (L-lactide) (PLLA, grade L130) and poly (D-lactide) (PDLA, grade D070) were received from Total Cobion PLA, Rayong, Thailand. The weight average molecular weight (M_w_) and number average molecular weight (M_n_) of the PLLA and PDLA were determined using gel permeation chromatography (GPC), with Tetrahydrofuran (THF) utilized as the eluent (see Table 1 for detail). Chloroform (CHCl_3_) was supplied by RCI Labscan Limited, Bangkok, Thailand. PEG with a molecular weight of 400 g/mol was purchased from Chemipan Corporation Co., Ltd., Bangkok, Thailand, and 35,000 g/mol from Sigma-Aldrich Co., Ltd. Singapore.

### 2.2. Sample Preparation

A solution of the PLLA/PDLA (50:50) blend was prepared by dissolving 1 g of material in 10 mL of the chloroform solvent. The blend solution was subjected to vigorous stirring for 3 h to ensure the thorough mixing and dissolution of the polymers. Afterward, PEG with different molecular weights (400 g/mol and 35,000 g/mol) was added to the PLLA/PDLA blend solution. PEG was incorporated in concentrations of 5%, 10%, and 20% by weight to create different blends. Following the addition of PEG, each mixture underwent an additional 3 h of vigorous stirring to achieve homogeneity. Subsequently, the prepared blend solutions were poured into Petri dishes, allowing the solvent (chloroform) to evaporate at room temperature over approximately 3 days. This process facilitated the formation of solid films as the solvent evaporated. Following evaporation, the films were vacuum-dried until a consistent weight was achieved. The final films had an approximate thickness of 0.5 mm. The sample codes and compositions of the PLA-PEG blend films utilized in this study are listed in Table 2.

### 2.3. Differential Scanning Calorimetry (DSC)

**Figure 2:**
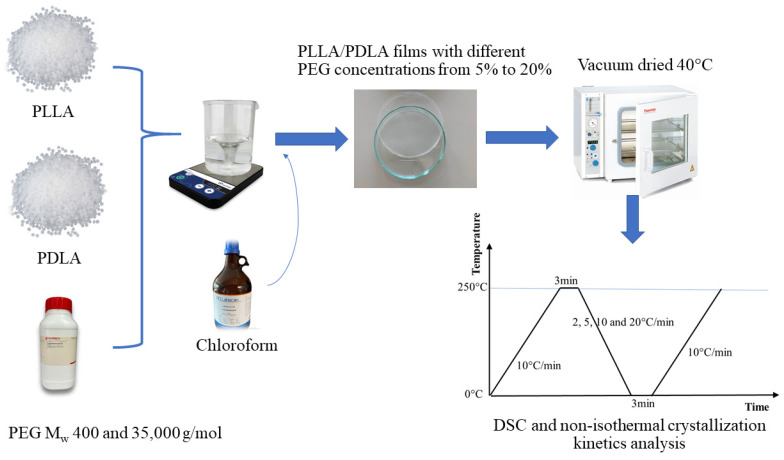
Schematic of the preparation process for fabricating PLLA/PDLA films with different PEG concentrations for non-isothermal crystallization kinetics analysis.

The thermal properties were assessed using differential scanning calorimetry (DSC) (Pyris Diamond DSC4000, Waltham, MA, USA) under a nitrogen gas flow of 20 mL/min. Prior to measurement, the instrument was calibrated with indium. The DSC data, including heat flow curves and thermograms, were recorded and analyzed to extract information about the thermal properties of the PLLA/PDLA and PEG blend films. The thermal procedures employed in different crystallization processes are shown as follows:

Samples weighing approximately 3–5 mg were packed in aluminum pans and subjected to non-isothermal melt crystallization. The heating process involved ramping from 0 to 250 °C at 10 °C/min, followed by a 3 min hold at 250 °C to eliminate thermal history. Subsequently, the samples were cooled to 0 °C at varying rates (2 °C/min, 5 °C/min, 10 °C/min, and 20 °C/min) with a 3 min hold to establish thermal equilibrium. Finally, the samples were reheated to 250 °C at a constant rate of 10 °C/min. Figure 2 illustrates the preparation process used to fabricate PLLA/PDLA films with different PEG concentrations for DSC analysis.

## 3. Results and Discussion

### 3.1. Effect of Different MWs of PEG on PLLA/PDLA Blends

#### 3.1.1. Cooling Cycle

**Figure 3:**
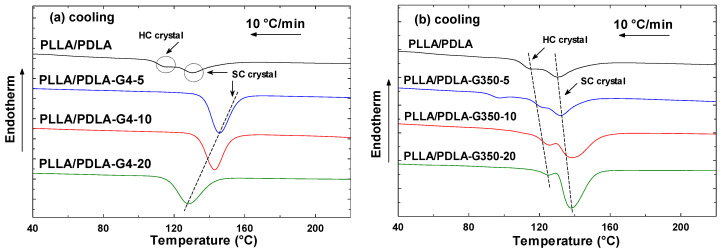
DSC cooling curves of (**a**) PLLA/PDLA-G4 and (**b**) PLLA/PDLA-G350 at a cooling rate of 10 °C/min.

**Figure 4:**
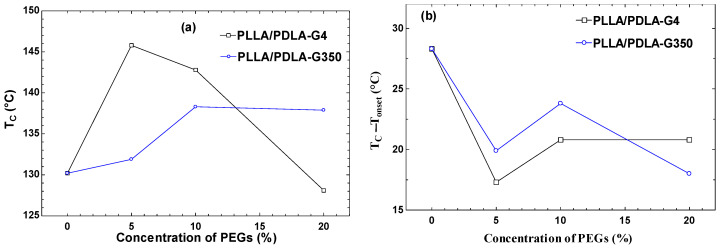
DSC-derived parameters during cooling: (**a**) crystallization temperature (T_c_), (**b**) the difference between onset crystallization temperature and T_c_ (T_onset_ − T_c_).

**Figure 5:**
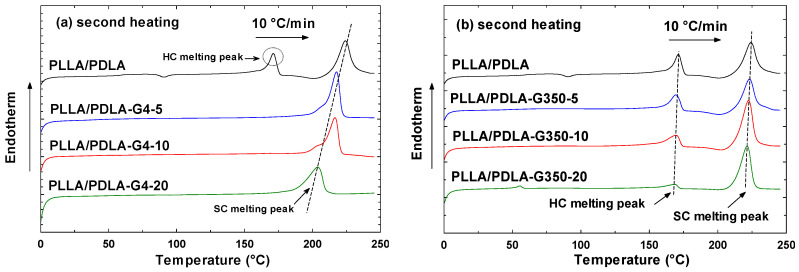
DSC second heating curves of (**a**) PLLA/PDLA-G4 and (**b**) PLLA/PDLA-G350 at a rate of 10 °C/min.

The data from the initial heating cycle inherently incorporate the influence of previous heating events. By excluding the temperature record from the initial heating scan, direct comparisons between different materials became viable through the information gleaned from the subsequent cooling cycle [32]. DSC thermograms of PLLA/PDLA-G4 and PLLA/PDLA-G350 (0–20% PEG concentrations) at a cooling rate of 10 °C/min are depicted in Figure 3a,b. Table 3 and Figure 4 present the obtained parameters, including the peak crystallization temperature and enthalpy (T_c_, H_c_), the difference between the initial and peak crystallization temperatures (T_onset_ − T_c_), and the crystallization half time (t_1/2_). As shown in Figure 3a, the presence of a single peak for different PEG contents with a molecular weight of 400 g/mol suggested the formation of SCs rather than HCs. This interpretation was supported by the reheating results shown in Figure 5a, where the melting temperature exceeds 200 °C, indicating the presence of SCs formed during the cooling process. Conversely, the addition of various PEG contents with a molecular weight of 35,000 g/mol (Figure 3b) displays two peaks, corresponding to the formation of both SCs and HCs.

In Figure 4a, at 5% G4 content (PLLA/PDLA-G4-5), T_c_ and ∆H_c_ were 145.8 °C and 77.5 J/g, respectively; subsequently, T_c_ experienced a sharp decline when increasing G4 content. Upon reaching 20% G4 (PLLA/PDLA-G4-20), Tc decreased to 128.1 °C. Conversely, in contrast to G4, the T_c_ of the PLLA/PDLA blended with G350 notably increased as the G350 content increased. The increase in G350 from 0% to 20% resulted in T_c_ increasing from 130.2 °C to 137.9 °C.

To explain why the crystallization temperature changes differently for PEG with M_w_ 400 and PEG with M_w_ 35,000 in PLLA/PDLA blends, we need to consider the effects of molecular weight, concentration, and the interaction of PEG with the polymer matrix. For PEG 400 g/mol, at low concentrations, PEG 400 acted as a plasticizer, which means it could penetrate and interact with the PLLA/PDLA chains, enhancing their mobility. This interaction disrupted the regular packing of the polymer chains, making it easier for them to move and align into a crystalline structure. This increased chain mobility could initially lead to a more efficient nucleation process, causing the crystallization temperature to rise. Beyond the 5% concentration, the excessive amount of PEG 400 could lead to phase separation and an over-plasticization effect. The high concentration of PEG 400 g/mol might create domains within the blend where PEG-rich and polymer-rich phases coexist. This phase separation hinders the crystallization process by creating heterogeneous regions and reducing the overall uniformity of the blend, thus lowering the crystallization temperature. The excess PEG might act as a diluent, making it harder for PLLA/PDLA chains to find each other and crystallize efficiently.

On the other hand, PEG 35,000 g/mol had a much higher molecular weight and lower solubility in PLLA/PDLA blends compared to PEG 400 g/mol. This higher-molecular-weight PEG could not penetrate the PLLA/PDLA matrix as effectively as PEG 400. Instead of acting as a plasticizer, PEG 35,000 tends to form a separate phase or large PEG-rich domains. These domains could act as nucleating agents, promoting crystallization at higher temperatures. Additionally, the high-molecular-weight PEG might slightly enhance the stiffness of the matrix, reducing the chain mobility and necessitating a higher temperature to achieve crystallization.

The total crystallization rate, calculated using T_onset_ − T_c_ (Figure 4b), indicated that smaller values indicate a faster total crystallization rate [33]. The T_onset_ − T_c_ value of the G4 and G350 blend stopped decreasing after 5% content and continued to increase, indicating that the overall crystallization rate of the blend was fastest at a 5% PEG content.

#### 3.1.2. Second Heating Cycle

Figure 5a,b depicts the DSC reheating curve and Table 4 presents the numerically computed data for the blends of PLLA/PDLA-G4 and PLLA/PDLA-G350. The thermograms exhibited cold crystallization and a melting transition throughout the reheating process.

**Figure 6:**
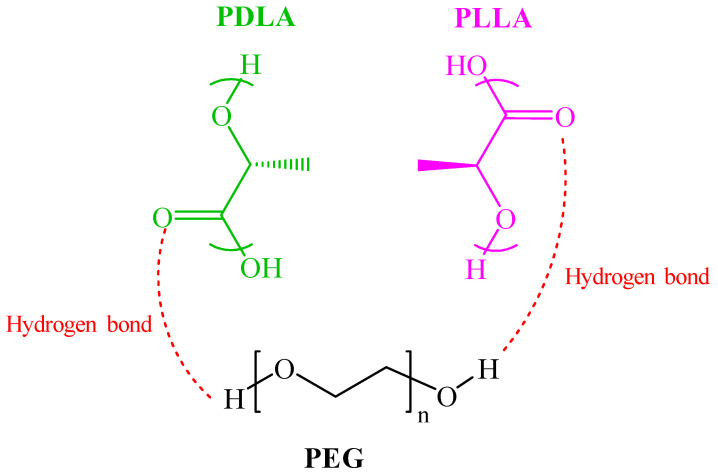
The molecular structures of PLLA, PDLA, and PEG.

As shown in Figure 5a, the neat PLLA/PDLA exhibited two melting peaks at around 170 °C and 220 °C, respectively, corresponding to the melting peak of homocrystals (HC) and stereocomplex crystals (SC). The stereocomplex structure moved the melting temperature higher that than of PLLA by ~50 °C [34]. This significant increase is due to hydrogen bonding interactions in the stereocomplex crystalline structure [35]. However, the blends with PEG 400 g/mol (G4) show the formation of complete SC crystallites without HC crystallites. Strong diffraction peaks of SC crystallites also indicate that SC crystallites are the exclusive crystalline species, and no HC crystallites form when a specific quantity of PEG 400 g/mol was utilized, ranging from 5–20%. These results clearly indicate that the presence of PEG had a significant impact on the competition between the growth of HCs and SCs. It was assumed that hydrogen bonds exist between polyethylene glycol (PEG) and polylactic acid (PLA). Su et al. [36] discussed that PEG, owing to its hydroxyl groups, can form hydrogen bonds with the carbonyl groups in PLA. Figure 6 illustrates the molecular structure of PLLA, PDLA, and PEG.

On the other hand, PLLA/PDLA-G350 (Figure 5b) exhibits two melting peaks at around 170 °C and 220 °C, respectively, corresponding to the melting peaks of HCs and SCs.

As shown in Figure 5 and Table 4, the cold crystallization curves observed during the second heating cycle of the PLLA/PDLA blends were accounted for in the degree of crystallinity analysis. The cold crystallization enthalpy (ΔH_cc_) was subtracted from the melting enthalpy (ΔH_m_) in the calculations to ensure an accurate determination of crystallinity. During the second heating cycle, cold crystallization peaks are usually associated with the recrystallization of amorphous regions into homocrystals [31]. Based on the findings from previous references [37,38,39], the crystallinity (X_c_) of the HCs and SCs in the PLLA/PDLA mixture is calculated using the following equation:

(1) $$X_{c}=\frac{∆H_{m}-∆H_{cc}}{∆H_{m}^{0}}\times 100\%$$

where ΔH_cc_ is the cold crystallization enthalpy, ΔH_m_ is the melting enthalpy of HC or SC crystallites as determined by the DSC measurement’s second heating curve, and $∆H_{m}^{0}$ represents the enthalpy of 100% HC or SC crystallites, which are 93 and 142 J/g for HC and SC crystallites, respectively [37,38,39].

**Figure 7:**
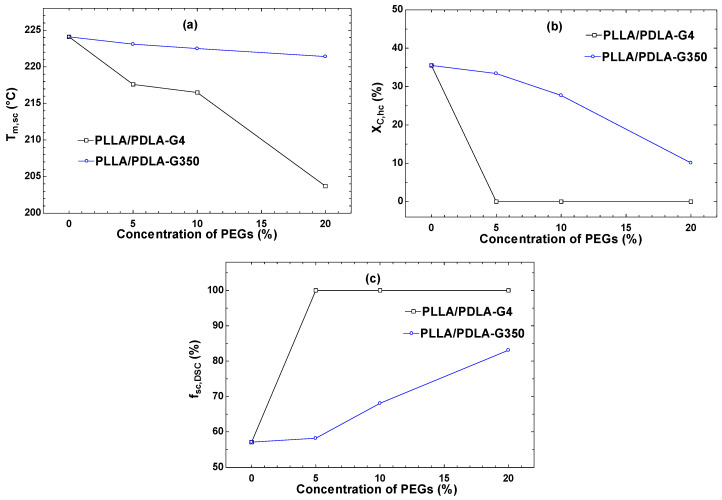
DSC derived parameter during the second heating (**a**) stereocomplex melting temperature (T_m,sc_), (**b**) the crystallinity of HC (X_c,hc)_, and (**c**) the relative fraction of SC crystallites of PLA-G4 and PLA-G350 evaluated from DSC measurement (f_sc,DSC_).

Figure 7a demonstrates the melting temperature of the stereocomplex crystallites (T_m,sc_). The addition of PEG 400 and 35,000 g/mol (0–20% concentrations) resulted in a continuous decrease in T_m,sc_ due to PEG acting as a plasticizer in the PLA matrix. As the PEG concentration increased, it interacted with the PLA chains, increasing their mobility and reducing the energy required for crystallization, thus lowering the melting temperature. Mohapatra et al. [40] reported similar findings, where PEG incorporation decreased the glass transition temperature (T_g_) and the melting temperature (T_m_). Additionally, Chalid et al. [41] showed that increasing PEG content reduced the crystallinity and peak intensity, leading to the structure becoming more amorphous.

Figure 7b illustrates the comparison of X_c,hc_ for HC crystallites in PLLA/PDLA blends with varying PEG concentrations. The inclusion of PEG significantly enhanced the ability of SC to crystallize. As the PEG concentration increased from 0 to 20 weight percent, the formation of HC crystallites was reduced, leading to a drastic decrease in X_c_(HC) with PEG incorporation. This observation suggests that PEG acted as an efficient nucleating agent in PLLA/PDLA blends, promoting the formation of SC crystallites.

To address this advancement, Figure 7c illustrates the relative fraction of SC crystallites (f_sc_), calculated by X_c_(SC)/[X_c_(HC) + X_c_(SC)]. For PLA, the value of f_sc_ remained at 57.1%. This value appeared to steadily increase with the addition of PEG, reaching complete stereocomplexation with 5–20 weight percent of PEG with a molecular weight of 400 g/mol. The findings suggest that PEG selectively influenced the formation of SC crystallites.

### 3.2. Effect of Different Cooling Rates on PLLA/PDLA Blends with PEG Incorporation

Polymer molding primarily follows a non-isothermal process, emphasizing the importance of comprehending the kinetic of non-isothermal crystallization to establish processing parameters and regulate product performance. Hence, the non-isothermal crystallization of PLLA/PDLA, PLLA/PDLA-G4-10, and PLLA/PDLA-G350-10 at various cooling rates was examined and theoretically analyzed.

#### 3.2.1. Cooling Cycle

**Figure 8:**
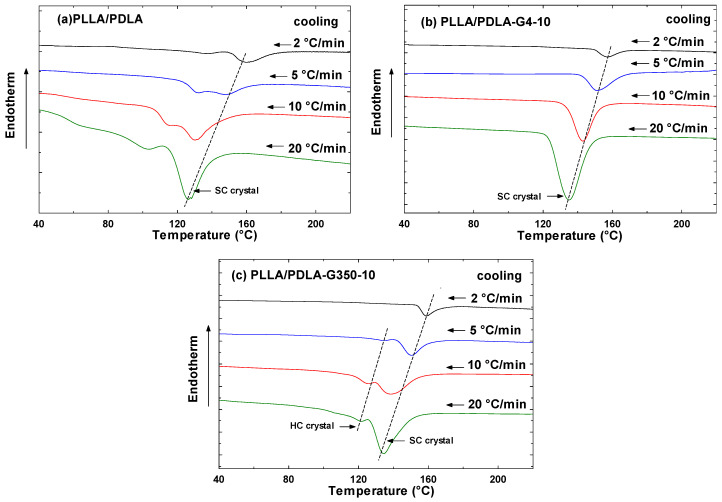
DSC cooling curves of PLLA/PDLA, PLLA/PDLA-G4-10, and PLLA/PDLA-G350-10 at various cooling rates (indicated on the curves).

Figure 8 depicts the DSC thermograms during cooling at a rate of 2, 5, 10, and 20 °C/min, while Table 5 summarizes the parameters determined from the DSC curves.

**Figure 9:**
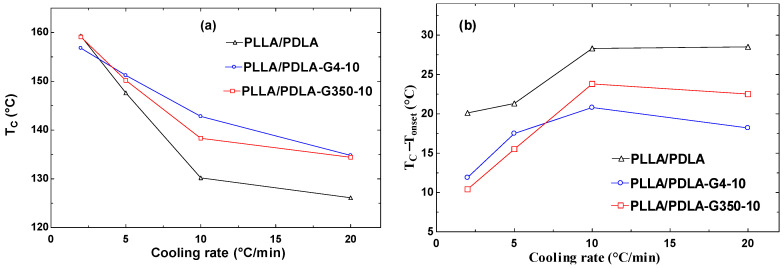
The effect of cooling rate on (**a**) T_c_ and (**b**) T_c_ − T_onset_ of PLLA/PDLA, PLLA/PDLA-G4-10, and PLLA/PDLA-G350.

The crystallization exotherms of PLLA/PDLA, PLLA/PDLA-G4-10, and PLLA/PDLA-G350-10 composites at four cooling rates are depicted in Figure 9. As expected for crystallization in a nucleation-controlled zone, the crystallization exotherm noticeably broadens and shifts to a lower temperature with increased cooling rates. With higher cooling rates, the temperature of the exothermic peaks for the PLLA/PDLA composite decreased from 156.3 °C to 126.1 °C. This occurred because there was less time available for the polymer to crystallize as the cooling rate escalated. Consequently, greater supercooling was required to initiate crystallization, leading to the widening of the exothermic peak. This aligns with the finding of Xiao et al. [42] who observed that with greater supercooling, nucleation occurred over a broader temperature range. As a result, crystallization did not occur at a single temperature but spanned a range of temperatures, causing the exothermic peak to broaden. Additionally, as shown in Figure 9, the interaction point occurred due to the interplay between different factors affecting the system’s behavior. The work by Hoffman and Weeks [43] on polymer crystallization kinetics indicated that interaction points occurred where nucleation and growth rates intersect under certain cooling conditions.

#### 3.2.2. Second Heating Cycle Following Non-Isothermal Crystallization

**Figure 10:**
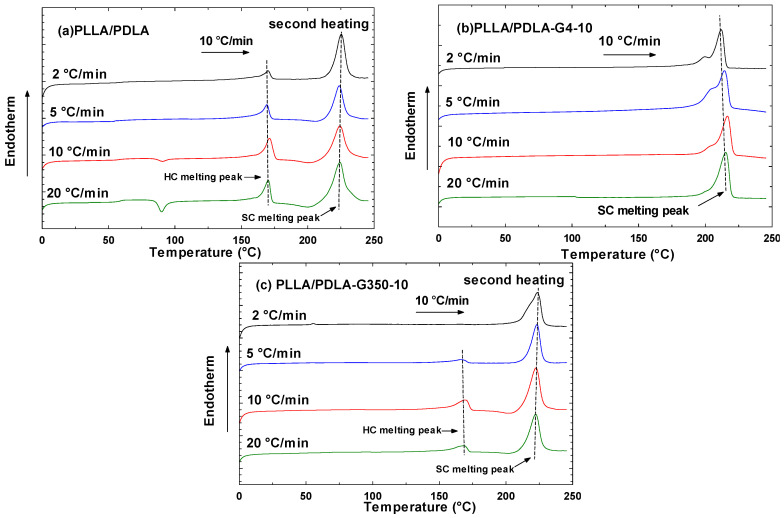
DSC second heating curves at a rate of 10 °C/min for PLLA/PDLA, PLLA/PDLA-G4-10, and PLLA/PDLA-G350-10 at various cooling rates (indicated on the curves).

While thermal history was encompassed in the data from the first heating cycle, the data from the second heating cycle enabled a direct comparison of the crystallization behavior of various materials devoid of thermal history [14]. The DSC second heating curve and associated cooling parameters at 2, 5, 10, and 20 °C/min (depicted in Figure 8) are presented in Figure 10 and Table 6.

As shown in Figure 10a, no cold crystallization peaks were observed in the second heating curves for neat PLLA/PDLA at cooling rates of 2 to 5 °C/min. This indicated that crystallization was fully complete at these low cooling rates. Additionally, two melting peaks appeared: one for HC around 172–173 °C and another for SC around 220 °C, with HC crystallites being more numerous than SC ones.

With the addition of PEG 400 g/mol, as shown in Figure 10b, the melting peak attributed to HC crystallization disappeared. This suggests that PEG 400 g/mol could penetrate and interact with the PLLA/PDLA chains, leading to favorable interactions with the carbonyls of PLLA or PDLA chains and encouraging the formation of SCs. Moreover, as the cooling rate decreased, the melting temperature of the stereocomplex crystals also decreased due to material degradation from prolonged heat exposure. The T_m,sc_ of PLLA/PDLA-G4-10 decreased from 215.3 °C at a 20 °C/min rate to 211.8 °C at a 2 °C/min rate. This reduction in T_m,sc_ at low cooling rates was attributed to the degradation of PLA.

Conversely, as illustrated in Figure 10c, PEG with a molecular weight of 35,000 g/mol, due to its significantly higher molecular weight and lower solubility in PLLA/PDLA blends compared to PEG 400 g/mol, was unable to penetrate the PLLA/PDLA matrix as effectively. Consequently, small HC formation was still observed.

#### 3.2.3. The Relationship between the Relative Crystallinity of PLLA/PDLA/PEG and Temperature and Time

The research by Xiao et al. [42] explored the non-isothermal crystallization behavior of poly(lactic acid) (PLA) with the addition of plasticizers and nucleating agents. The relative crystallinity (X_T_) at any given temperature (T) can be determined using the following equation:


(2) $$X_{T}=\frac{\int_{T_{0}}^{T}({dH}_{c}/dT)dT}{\int_{T_{0}}^{T_{\infty }}({dH}_{c}/dT)dT}$$

**Figure 11:**
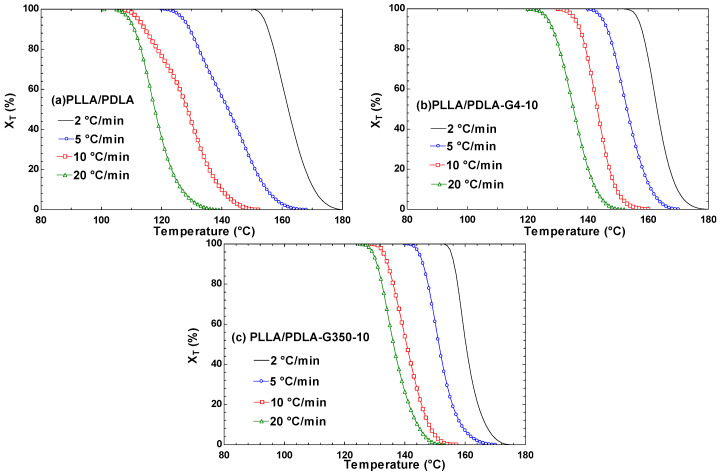
The relative crystallinity (X_T_) versus crystallization temperature (T) for PLLA/PDLA, PLLA/PDLA-G4-10, and PLLA/PDLA-G350-10 at various cooling rates.

In Equation (2), T_0_ and T_∞_ represent the beginning and ending temperatures of crystallization, respectively. The exothermic enthalpy of crystallization over a small temperature range, dT, is denoted by dH_c_ [42]. Figure 11a–c depicts the X_T_T curves for PLLA/PDLA and PEG blends. The crystallinity at various cooling rates exhibited an inverted S-shape, indicating distinct stages of crystallization. Initially, the nucleation process was relatively slow, followed by predominant crystal growth during the intermediate stage of crystallization. Subsequently, crystallinity increased rapidly before gradually stabilizing in the later stage as crystals continued to grow. Moreover, Figure 11 illustrates that within the same sample, decreasing the cooling rate led to earlier attainment of complete crystallization. This observation suggests that undercooling phenomena occurred when molecules lacked sufficient time to undergo crystallization as the cooling rate increased.

**Figure 12:**
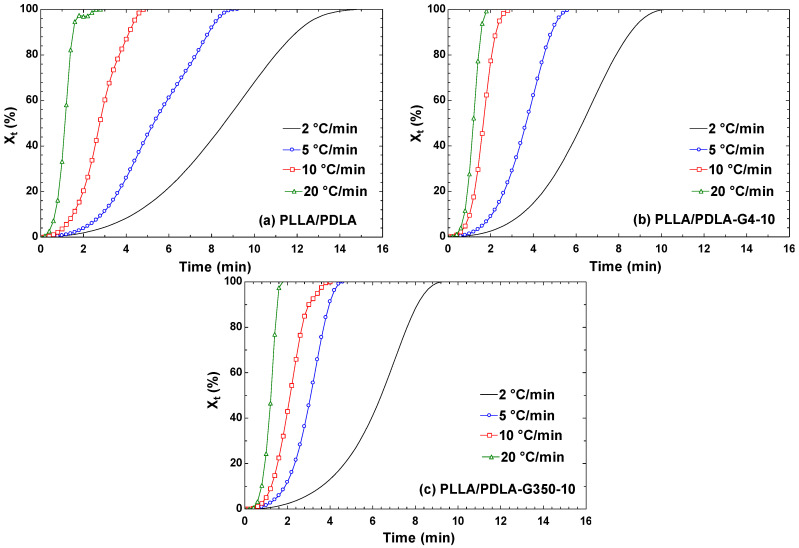
The relative crystallinity (X_t_) versus crystallization time (t) for PLLA/PDLA, PLLA/PDLA-G4-10, and PLLA/PDLA-G350-10 at various cooling rates.

X_T_ data can be transformed into X_t_ using the relationship shown below [44]:

(3) $$X_{t}=\frac{T_{0}-T}{D}$$

where T is the temperature at crystallization time t and D represents the cooling rate. In Figure 12a–c, under low cooling rates (2 °C/min), the impact of cooling rate delay on the crystallization of the PLLA/PDLA/PEG blend became evident, with the X_t_-t curve displaying a distinct S-shape. Conversely, at higher cooling rates, the molten PLA swiftly transitions into a glassy state, leading to a linear X_t_-t curve. Consequently, the time required for crystallization completion was shortened. Additionally, the ascent of the curve significantly decelerated during the later stages of crystallization. This phenomenon arose because as crystals enlarge, they begin to interact, forming grain boundaries that impede the continuous growth of crystals and eventually halt crystallization. Furthermore, from Figure 12, it was observed that PLLA/PDLA blended with PEG 35000 (Figure 12c) and PEG 400 (Figure 12b) exhibited shorter crystallization times compared to pure PLLA/PDLA (Figure 12a).

At a cooling rate of 2 °C/min, PLLA/PDLA fully crystallized in approximately 15 min, whereas PLLA/PDLA-G4-10 and PLLA/PDLA-G350-10 reached full crystallization in just 9–10 min. When the cooling rate increased to 5 °C/min, PLLA/PDLA-G4-10 and PLLA/PDLA-G350-10 completed crystallization within 4–5 min, showing that the lower cooling rates prolonged crystallization times. This suggests that the addition of PEG to PLLA/PDLA materials enhances their susceptibility to crystallization.

### 3.3. Non-Isothermal Crystallization Kinetics of PLLA/PDLA/PEG Blends

Polymer processing is a multifaceted endeavor influenced by factors such as the molecular structure of the polymer, its chain segments, and the processing temperature. A thorough understanding of how crystal structures in polymers react to temperature changes can be gained by exploring non-isothermal crystallization.

The Avrami equation serves as a crucial theoretical model in the examination of the crystallization kinetics of polymer materials [45], formulated as shown in Equation (4).


(4) $$1-X_{t}=\exp(-Z_{t}\times t^{n})$$

In this equation, X_t_ represents the relative crystallization fraction; Z_t_ denotes a non-isothermal crystallization rate constant linked to both the nucleation rate of polymers and the growth rate of crystals; t signifies time; and *n* stands for the Avrami index, which indicates the polymer’s crystallization mechanism. By modifying the Avrami equation, we derive Equation (5) as illustrated below:


(5) $$\mathrm{lg}\left[-\ln\left(1-X_{t}\right)\right]=nlgt+lgZ_{t}$$

**Figure 13:**
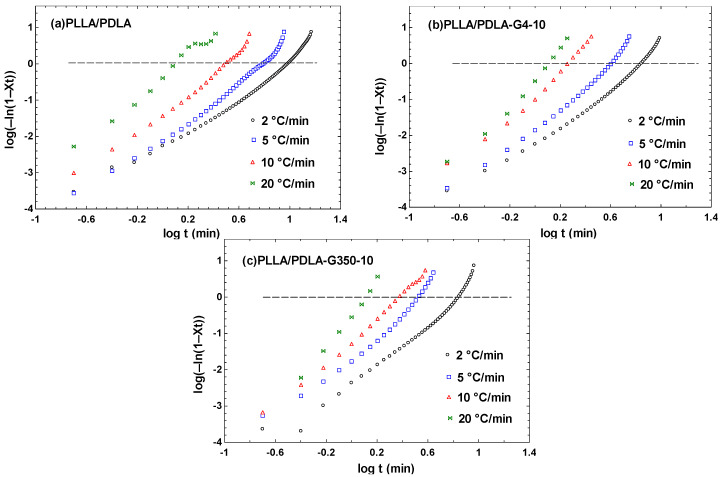
Fitting curves of lg[−ln(1 − X_t_)] with respect to lgt for PLLA/PDLA, PLLA/PDLA-G4-10, and PLLA/PDLA-G350-10 at various cooling rates.

Plotting lgt against lg[−ln(1 − x)] yields Figure 13. Examining the curve’s behavior under different cooling rates revealed two distinct stages: the primary and secondary crystallization stages. The secondary stage showed a more complex process, causing deviations from linearity. Linear regression analysis of the primary crystallization stage yielded a slope representing *n*, with Z_t_ calculated from the intercept. Table 7 lists the *n* and Z_t_ values at different cooling rates. The data indicate that both low- and high-molecular-weight PEG had higher *n*-values compared to neat PLLA/PDLA blends, suggesting that PEG promoted crystallization in PLLA/PDLA blends.

### 3.4. Non-Isothermal Crystallization Activation Energy

The activation energy (ΔE) [46] of non-isothermal crystallization can be calculated using the equation formulated by Takhor. This equation accounts for the change in peak temperature (T_c_) with the cooling rate (D), where R is the gas constant with a value of 8.314 J/(mol∙K). The formulation is given as:

(6) $$\ln\left(D\right)=\frac{-∆E_{T}}{R}(\frac{1}{T_{c}})$$

**Figure 14:**
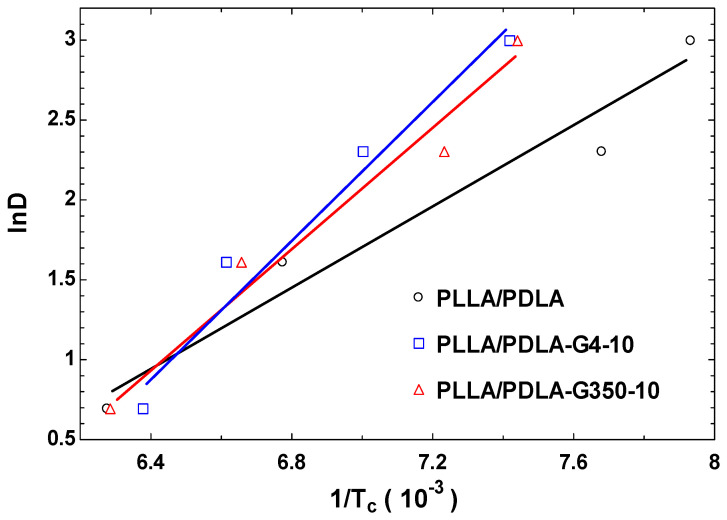
Plots of ln D versus 1/T_c_ using the Takhor equation.

After substituting D and T_c_ from Table 5 into the equation, the natural logarithm of D (ln(D)) was plotted against 1/T_c_ to obtain the data points in Figure 14. The non-isothermal crystallization activation energies (ΔE_T_) can be derived from the slope, and these values are listed in Table 8. According to Zhou and Cao [47], their study on non-isothermal crystallization kinetics of glass fiber-reinforced PA66 composites revealed important insights into the crystallization behavior of such composites under varying cooling rates. Their findings highlighted that the activation energy values are critical in understanding the crystallization process, which can significantly influence the thermal and mechanical properties of the composites.

The analysis of Figure 14 and Table 8 reveals noticeable alterations in the activation energy of non-isothermal crystallization upon the introduction of PEG to the PLLA/PDLA blends. Specifically, adding PEG400 and PEG35000 led to a marked decrease in the activation energy for non-isothermal crystallization. These findings suggest that the incorporation of PEG had a substantial influence on the crystallization behavior, potentially enhancing the crystallization of PLLA/PDLA/PEG blends. Future investigations may explore a wider range of PEG contents to further elucidate these effects.

## 4. Conclusions

Our study has identified an effective method to enhance the melt crystallization of stereocomplex crystals (SCs) within PLLA/PDLA blends by introducing PEG into the system. This enhancement, particularly noticeable with higher concentrations and lower molecular weight (M_w_) of PEG, improved the formation of SC crystallites while minimizing the occurrence of homocrystallites (HCs). In this work, various amounts of PEG with molecular weights of 400 g/mol and 35,000 g/mol were added to PLLA/PDLA 50/50 at concentrations ranging from 5% to 20%. DSC test results showed that adding 5% PEG with a molecular weight of 400 g/mol resulted in complete SC-PLA formation, whereas adding 5% PEG with a molecular weight of 35,000 g/mol resulted in only 58.2% SC-PLA formation. The kinetics of non-isothermal crystallization were investigated using the Avrami technique, revealing higher *n* values for PEG-containing mixtures compared to neat PLLA/PDLA blends, confirming that PEG promoted crystallization in these blends. Moreover, the activation energy for non-isothermal crystallization indicated that the mixture containing low-molecular-weight PEG exhibited the greatest advantage, with an activation energy of 17.6 kJ·mol^−1^. These findings highlight the practical implications of developing PLA-based materials with enhanced performance, making them more suitable for a wider range of applications, including packaging, textiles, and biomedical devices.

## Figures and Tables

**Table 1:** Material characterization.

	PLLA	PDLA
Code	L130	D070
Optical Purity	≥99% (L-isomer)	≥99% (D-isomer)
Weight-average molecular weight (Mw) (GPC) *^1^	1.68 × 10^5^	0.87 × 10^5^
M_w_/M_n_	2.02	1.73

*^1^ The GPC column was calibrated using the standard polystyrene with tetrahydrofuran (THF) as eluent at room temperature.

**Table 2:** Composition of PLLA/PDLA (50/50) with added PEGs.

Sample Code	Average MW (M_n_) (g/mol) of PEG	Concentration of PEGs (wt%)	Composition (wt%)		
			PLLA	PDLA	PEG
PLLA/PDLA	-	0	50	50	0
PLLA/PDLA-G4-5	400	5	47.5	47.5	5
PLLA/PDLA-G4-10	400	10	45	45	10
PLLA/PDLA-G4-20	400	20	40	40	20
PLLA/PDLA-G350-5	35,000	5	47.5	47.5	5
PLLA/PDLA-G350-10	35,000	10	45	45	10
PLLA/PDLA-G350-20	35,000	20	40	40	20

**Table 3:** Differential scanning calorimetry (DSC) data derived from cooling thermograms of PLLA/PDLA-G4 and PLLA/PDLA-G350.

Samples	∆H_c_	T_c_	T_onset_ − T_c_	t_1/2_
	(J/g)	(°C)	(°C)	(min)
PLLA/PDLA	−48.4	130.2	28.3	5.6
PLLA/PDLA-G4-5	−77.5	145.8	17.3	3.4
PLLA/PDLA-G4-10	−68.2	142.8	20.8	4.1
PLLA/PDLA-G4-20	−67.3	128.1	20.8	4.1
PLLA/PDLA-G350-5	−51.6	131.9	19.9	3.9
PLLA/PDLA-G350-10	−76.5	138.3	23.8	4.7
PLLA/PDLA-G350-20	−66.0	137.9	18.0	3.6

**Table 4:** Differential scanning calorimetry (DSC) data derived from second heating thermograms of PLLA/PDLA-G4 and PLLA/PDLA-G350.

Samples	Cold Crystallization		Homocrystal Melting (HC)		Stereocomplex Melting (SC)		X_c,hc_	X_c,sc_	f_sc,DSC_
	∆H_cc_	T_cc_	∆H_m,hc_	T_m,hc_	∆H_m,sc_	T_m,sc_	(%)	(%)	(%)
	(J/g)	(°C)	(J/g)	(°C)	(J/g)	(°C)			
PLLA/PDLA	−3.6	90.5	33.0	171.1	67.1	224.1	31.6	47.3	59.9
PLLA/PDLA-G4-5	-	-	-	-	74.7	217.6	0.0	52.6	100.0
PLLA/PDLA-G4-10	-	-	-	-	62.3	216.5	0.0	43.9	100.0
PLLA/PDLA-G4-20	-	-	-	-	67.0	203.7	0.0	47.2	100.0
PLLA/PDLA-G350-5	-	-	31.0	169.1	66.1	223.1	33.4	46.5	58.2
PLLA/PDLA-G350-10	-	-	25.7	169.5	84.1	222.5	27.7	59.2	68.1
PLLA/PDLA-G350-20	-	-	9.4	168.3	70.8	221.4	10.1	49.8	83.1

**Table 5:** Cooling parameters of PLLA/PDLA, PLLA/PDLA-G4-10, and PLLA/PDLA-G350-10 at various cooling rates.

Samples	Cooling Rate (°C/min)	∆H_c_	T_c_	T_onset_ − T_c_
		(J/g)	(°C)	(°C)
PLLA/PDLA	2	−70.4	159.3	20.1
	5	−52.3	147.6	21.3
	10	−48.4	130.2	28.3
	20	−34.6	126.1	28.5
PLLA/PDLA-G4-10	2	−60.9	151.8	11.9
	5	−72.3	151.2	17.5
	10	−68.2	142.8	20.8
	20	−70.3	134.8	18.2
PLLA/PDLA-G350-10	2	−67.4	159.1	10.4
	5	−58.6	150.2	15.5
	10	−76.5	138.3	23.8
	20	−59.8	134.4	22.5

**Table 6:** Second heating parameters at 10 °C/min of PLLA/PDLA, PLLA/PDLA-G4-10, and PLLA/PDLA-G350-10 at various cooling rates.

Samples	Cooling Rate (°C/min)	Cold Crystallization		Homocrystal Melting (HC)		Stereocomplex Melting (SC)	
		∆H_cc_	T_cc_	∆H_m,hc_	T_m,hc_	∆H_m,sc_	T_m,sc_
		(J/g)	(°C)	(J/g)	(°C)	(J/g)	(°C)
PLLA/PDLA	2	-	-	13.5	170.1	64.9	225.2
	5	-	-	21.3	169.2	52.6	223.6
	10	−3.6	90.5	33.0	171.1	67.1	224.1
	20	−13.0	89.9	30.0	170.1	85.6	224.3
PLLA/PDLA-G4-10	2	-	-	-	-	59.6	211.8
	5	-	-	-	-	81.6	214.3
	10	-	-	-	-	62.3	216.5
	20	-	-	-	-	68.5	215.3
PLLA/PDLA-G350-10	2	-	-	0.6	164.3	74.3	223.6
	5	-	-	8.4	166.0	60.0	223.2
	10	-	-	25.7	169.5	84.1	222.5
	20	-	-	17.2	168.5	70.1	222.3

**Table 7:** Crystallization kinetics parameters of PLLA and PLLA/PDLA/PEG blends.

	D (°C/min)	Main Crystallization Stage	
		*n*	Z_t_
PLLA/PDLA	2	2.49	0.0055
	5	3.39	0.0075
	10	3.44	0.0367
	20	2.66	0.4033
PLLA/PDLA-G4-10	2	2.79	0.0057
	5	3.60	0.0139
	10	4.00	0.0999
	20	4.75	0.3235
PLLA/PDLA-G350-10	2	3.44	0.0044
	5	3.97	0.0168
	10	3.54	0.0511
	20	6.13	0.2794

**Table 8:** Activation energy of PLLA/PDLA/PEG blends.

Sample	∆H_T_ (KJ·mol^−1^)
PLLA/PDLA	−10.34
PLLA/PDLA-G4-10	−17.6
PLLA/PDLA-G350-10	−15.23

## Data Availability

The original contributions presented in the study are included in the article, further inquiries can be directed to the corresponding author.
